# The Dual Diverse Dynamic Reversible Effects of Ankaferd Blood Stopper on EPCR and PAI-1 Inside Vascular Endothelial Cells With and Without LPS Challenge

**DOI:** 10.5152/tjh.2011.41

**Published:** 2012-12-05

**Authors:** Afife Karabıyık, Erkan Yılmaz, Şükrü Güleç, İbrahim Haznedaroğlu, Nejat Akar

**Affiliations:** 1 Ankara University, Department of Pediatric Molecular Genetics, Ankara, Turkey; 2 Hacettepe University, Department of Hematology, Ankara, Turkey

**Keywords:** Ankaferd, EPCR, PAI-1, LPS, HUVEC

## Abstract

**Objective:** Ankaferd blood stopper (ABS) is comprised of a mixture of the plants Thymus vulgaris, Glycyrrhiza glabra, Vitis vinifera, Alpinia officinarum, and Urtica dioica. ABS is used as a topical hemostatic agent due to its antihemorrhagic effect, yet its hemostatic mechanism of action remains to be investigated. ABS does not affect the levels of coagulation factors II, V, VII, VIII, IX, X, XI and XII. The aim of this study was to investigate the effects of ABS on endothelium and immune response. As such, we evaluated changes in endothelial cell protein C receptor (EPCR) and plasminogen activator inhibitor type-1 (PAI-1) expression inside human umbilical vein endothelial cells (HUVECs) in the presence and absence of lipopolysaccharides (LPSs).

**Material and Methods:** We exposed HUVECs to 10 μL and 100 μL of ABS for 5 min, 25 min, 50 min, 6 h, and 24 h. Additionally, 10 μg mL–1 of LPS was administered for 1 h to observe the effects of LPS challenge on HUVECs, and then the cells were treated with ABS for 5 min, 25 min, 50 min, and 6 h to observe the effects of ABS on HUVECs. Total RNA was isolated from HUVECs and then the level of expression of EPCR and PAI-1 mRNA was measured.

**Results:** Cells were microscopically observed to arise from the surface and adhere to each other following the administration of ABS to HUVECs. Additionally, after 24 h the cells had normal growth and physiology, which suggests that the adhesive cellular effects of ABS might be reversible. ABS had a negative effect on EPCR and PAI-1 expression; the effect in response to 100 µL was greater than that to 10 µL. EPCR and PAI-1 expression increased over time in response to LPS and 10 µL of ABS. EPCR and PAI-1 expression was very low during the first hour of exposure to LPS and 100 µL of ABS, but after 6 h increased to levels similar to those observed in response to LPS and 10 µL of ABS.

**Conclusion:** It was observed that ABS had dual diverse dynamic reversible effects on EPCR and PAI-1 expression in HUVECs, which were dependent on dose and concentration. ABS might play a role in numerous cellular mechanisms, in addition to having hemostatic effects.

**Conflict of interest:**None declared.

## Abstract (tr)

**Amaç:** Ankaferd, Thymus vulgaris, Glycyrrhiza glabra, Vitis vinifera, Alpinia officinarum ve Urtica dioica bitkilerindenoluşan bir karışımdır. Kanamayı durdurucu etkisinden dolayı topikal hemostatik ajan olarak kullanılmaktadır.Hemostatik çalışma mekanizması araştırılmaktadır. Koagülasyon faktörleri II, V, VII, VIII, IX, X, XI ve XII seviyeleriAnkaferd’den etkilenmemektedir. Çalışmamızda Ankaferd’in endotel üzerindeki ve immün yanıttaki etkisini araştırmakamacıyla, İnsan Umbilical Ven Endotel Hücreleri (HUVEC) kullanılarak Endotelyal Protein C Reseptörü (EPCR) vePlazminojen Aktivatör İnhibitör (PAI-1) gen ekspresyonları üzerindeki etkisi araştırılmış ve farklı doz ve zamanlardaoluşturduğu değişikliklerin incelenmesi, lipopolisakkarit (LPS) muamelesinden sonra EPCR ve PAI-1 ekspresyonlarıüzerindeki olası etkisinin gösterilmesi amaçlanmıştır.

**Gereç ve Yöntemler:** HUVEC’lere 10 µl ve 100 µl konsantrasyonlarda Ankaferd 5 dk, 25 dk, 50 dk, 6 saat ve 24saat süreyle uygulanmıştır. Ayrıca immün yanıt üzerindeki etkisinin incelenebilmesi için 10 µg/ml LPS ile 1 saatlikmuamelenin ardından hücreler 10 µl ve 100 µl Ankaferd’le birlikte 5 dk, 25 dk, 50 dk ve 6 saatlik sürelerde maruzbırakılmıştır. HUVEC hücrelerinden RNA izolasyonu yapılmış; EPCR ve PAI-1 gen ekspresyonları incelenmiştir.

**Bulgular:** Yapılan mikroskobik incelemede Ankaferd uygulamasıyla hücrelerin yüzeyden kalkıp toplanarak birbirlerineyapıştıkları ve 24 saatin içerisinde büyüme ve gelişmelerinin normale döndüğü gözlenmiştir. Bu, Ankaferd’in etkisininzamanla azaldığı anlamına gelebilir. Ankaferd muamelesiyle başlangıçta gözlenen EPCR ve PAI-1 ekspresyonundakideğişim zamanla azalmıştır. Ayrıca ekspresyonlardaki hızlı değişim göz önüne alındığında Ankaferd’in artankonsantrasyonlarda etkisini daha da fazla gösterdiği söylenebilir. Böylece Ankaferd’in EPCR ve PAI-1 üzerinde hemdoz, hem de zamana bağlı etkisi belirlenmiştir. LPS muamelesi sonrasında da hücreler üzerinde Ankaferd benzer bir etkigöstermekte, doz arttıkça daha da etkili hale gelmekte ve zamanla etkisini yitirmeye başlamaktadır.

**Sonuç:** Ankaferd’in HUVEC’lerde EPCR ve PAI-1 ekspresyonu üzerinde doz ve konsantrasyona bağımlı etkisinin varlığısaptanmış olup, hemostatik etkileri yanısıra hücresel birçok mekanizmayı da etkileyebileceği düşünülmektedir.

## INTRODUCTION

Ankaferd blood stopper (ABS) is a unique medicinal plant extract mixture, which has been historically used as a hemostatic agent in Turkish folk medicine [1,2]. ABS is comprised of a standardized mixture of the plants Thymus vulgaris, Glycyrrhiza glabra, Vitis vinifera, Alpinia officinarum, and Urtica dioica. Following numerous preclinical experiments [2,3,4,5,6,7] and a clinical phase I study [8], ABS was approved for use in Turkey as a medicinal product for the management of external hemorrhage, the post-dental surgery period, and bleeding refractory to conventional anti-hemorrhagic agents [9,10,11,12,13,14,15,16]. 

The basic mechanism of action of ABS is formation of an encapsulated protein network that provides focal attachment points for very rapid (<1 s) vital erythrocyte aggregation—known as the hemostatic ABS-web [1,17]. ABS-induced protein network formation with blood cells—particularly erythrocytes—covers the primary and secondary hemostatic system without disturbing individual coagulation factors [1,5,7,17]. ABS also has antiinfection and anti-neoplastic effects [18,19,20]. The distinct important molecular components of the ABS-induced hemostatic network involve vascular endothelium, proteins, and blood cells [17,21,22,23,24]. Endothelial protein C receptor (EPCR) plays a role in numerous hemostatic, vascular, and immunological actions [25,26,27,28,29,30,31,32,33]. Likewise, plasminogen activator inhibitor type-1 (PAI-1) is a very important biological mediator of fibrinolysis, infection, neoplasia, obesity, and wound healing [34,35,36]. 

The aim of the present study was to examine the intracellular effects of ABS on EPCR and PAI-1 expression in human umbilical vein endothelial cells (HUVECs), as these molecules may be novel administrators at the center of ABS-induced pleiotropic effects. Lipopolysaccharides (LPSs) are large molecules that act as endotoxins and elicit strong immune responses within the vascular system [37]. LPS challenge is the process of exposing a biological environment to an LPS that may behave as a toxin in order to observe immunological and hemostatic responses. Hence, the present study aimed to investigate the effects of ABS— with and without LPS challenge—on HUVECs, based on changes in EPCR and PAI-1 expression.

## MATERIALS AND METHODS

ABS (10 μL and 100 μL) was administered to HUVECs (75 cm^2^ and ~75% fullness) for 5 min, 25 min, 50 min, 6 h, and 24 h. Nuclei were isolated from HUVECs, and the level of expression of EPCR and PAI^-1^ was determined using a Roche LightCycler 1.5 (Basel, Switzerland). Fluorescence- marked primers were used to analyze EPCR and PAI^-1^ expression. Water with a pH of 2 (likely to be similar to the pH of ABS) was used as a control. In addition, to observe the effects of LPS challenge on HUVECs and those of ABS on HUVECs 10 μg mL^–1^ of LPS (Sigma, Germany) was administered for 1 h to the test platform. Then, the cells were exposed to ABS for 5 min, 25 min, 50 min, and 6 h, so as to measure ABS-induced changes in EPCR and PAI^-1^ expression in relation to LPS. All experiments were repeated at least 2 times. Statistical analysis was based on two-way ANOVA with Bonferroni post test using Graph- Pad Prism v.5.0 (GraphPad Software, San Diego California, USA, http://www.graphpad.com).

## RESULTS

**Figure 1:**
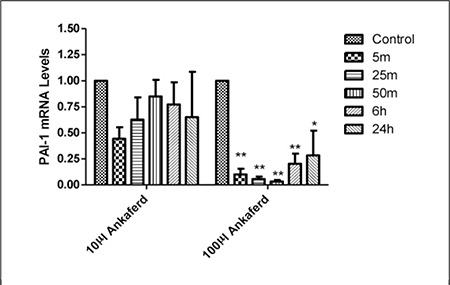
The effect of 10 μL and 100 μL of ABS on PAI-1mRNA expression (*P < 0.05 and **P < 0.01). Error bars represent Means ± SD

**Figure 2:**
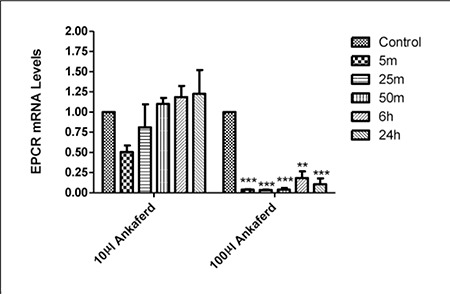
The effect of 10 μL and 100 μL of ABS on EPCR mRNA expression (**P < 0.01 and ***P < 0.001).

Cells were microscopically observed to arise from the plastic surface and adhere to each other in response to ABS administration to HUVECs. Additionally, after 24 h the cells had normal growth and physiology, which suggests that the adhesive cellular effects of ABS might be reversible. PAI^-1^ and EPCR expression was negatively affected by 24 h of exposure to 100 µL of ABS (Figures 1 and 2), but not by exposure to 10 µL of ABS. These findings indicate that the dose-dependent effects of ABS rely on PAI^-1^ and EPCR gene expression. 

**Figure 3:**
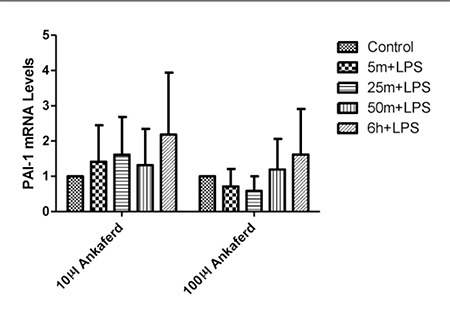
The effect of LPS, and 10 µL and 100 µL of ABS onPAI-1 mRNA expression. Error bars represent Means ± SD.

**Figure 4:**
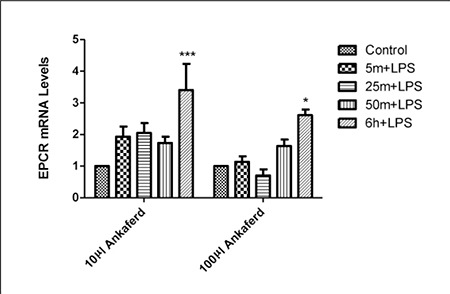
The effect of LPS, and 10 µL and 100 µL of ABS on EPCR mRNA expression (*P < 0.05 and ***P < 0.001). Error bars represent Means ± SD.

When LPS only was administered to HUVECs, EPCR and PAI^-1^ expression was higher than in HUVECs not exposed to LPS (data not shown). When LPS, and 10 µL and 100 µL of ABS were administered, the level of PAI^-1^ expression was stable and similar to that in the control after 6 h (Figure 3), whereas EPCR expression was very low during the first h of exposure, but increased after 6 h(Figure 4).

## DISCUSSION

ABS exhibited dual diverse dynamic reversible effects on EPCR and PAI^-1^ expression in HUVECs. The observed immediate increase in the level of expression of pro-hemostatic PAI^-1^ and down-regulation of anti-coagulant EPCR following exposure to ABS are compatible with previous reports of ABS’s sudden anti-hemorrhagic effect [3,4,5,6,7] and clinical backgrounds [8,9,10,13,14,15,20]. The topical hemostatic efficacy of ABS has been previously tested in animals with normal [4,6] and dysfunctional hemostasis [5,7]. Experimental studies have led to the preclinical stage of this hemostatic product’s development. Short-term oral systemic administration of ABS in rabbits was reported to be hematologically and biochemically safe, as acute mucosal toxicity, hematotoxicity, hepatotoxicity, nephrotoxicity, and biochemical toxicity were not observed during the short-term follow-up [3]. Those preclinical results reflect a starting point to search any possible systemic confounding effect of ABS when applied to internal topical surfaces. 

The use of ABS as a hemostatic agent for treating external hemorrhages and for dental treatment in humans is thie first indication the ABS is safe and efficacious in humans [8]. A phase I double-blind, randomized crossover placebo-controlled clinical study with a 5-d washout period between cross-over periods that included healthy volunteers reported that ABS was safe. Physiological cellbased coagulation was clinically obtained in response to topical ABS administered for the prevention and treatment of bleeding associated with many distinct clinicopathological states [8,9,10,13,14,15,20]. The ABS-induced hemostatic network consists of distinct important molecular components. Vital erythroid aggregation occurs in the spectrin, ankyrin, and actin proteins in red blood cell membranes. Essential erythroid proteins (ankyrin recurrent and FYVE bundle containing protein 1, spectrin alpha, actin-depolymerization factor, actin-depolymerizing factor, LIM bundle and actin-binding subunit 1 isoform a, LIM bundle and actin-binding subunit 1 isoform b, NADP-dependent malic enzyme, NADH dehydrogenase [ubiquinone] 1 alpha subcomplex, mitochondrial NADP [+]-dependent malic enzyme 3, ribulose bisphosphate carboxylase large chain, and maturase K) and the required ATP bioenergy (ATP synthase, ATP synthase beta subunit, ATP synthase alpha subunit, ATP-binding protein C12, TP synthase H+ transporter protein, ADF, and alpha^-1^,2-glycosyltransferase ALG10-A) are included in the ABS protein library. ABS also upregulates the GATA/ FOG transcription system, affecting erythroid functions and urotensin II [21,23,24]. 

The initial vascular dynamic response to ABS is vasoconstriction, whereas the late response is vasodilatation [22]. The clinical control of critical bleeding states associated with deficiencies of either primary or secondary hemostasis has been previously examined [13, 38]. Patients with bleeding diathesis that could not be controlled with standard anti-hemorrhagic methods were successfully treated with topical ABS [9,13,16,38]. The present study’s results provide additional evidence that ABS might also affect vascular anticoagulant (namely EPCR) and antifibrinolytic (namely PAI^-1^) pathways in a balanced way to regulate hemostasis, even in individuals with clotting defects. 

In addition to its hemostatic activity, ABS might also inhibit the growth of bacteria [19]. The anti-infection activity of ABS may bolster its current clinical use, as it inhibits the growth of bacteria in the region in which it is applied primarily for its hemostatic activity, such as infected wounds. The antimicrobial activity of ABS was tested against many pathogens, including A. baumannii, E. coli, K. pneumonia, P. aeruginosa, Enterobacter spp., Stenotrophomonas maltophilia, methicillin-resistant coagulase-negative Staphylococcus, vancomycin-susceptible Enterococcus, and VRE. ABS was reported to exhibit antibacterial activity against several gram-positive and gramnegative food and human pathogens [18]; however, the mechanism of action of ABS’s anti-infection effect is currently unknown. EPCR is an important molecule involved in the regulation of biological responses to severe infection [25,39]. LPS-induced endotoxemia requires the enzymatic active site of EPCR and PAR^-1^ [39]. In the present study ABS up-regulated the expression of EPCR and PAI^-1^ in the presence of LPS. The anti-infection effect of ABS might be related to its hemostatic effects on distinct steps of coagulation and vascular endothelium. 

Preliminary observations indicated that ABS might have wound healing effects in different clinical states, such as oral infections, rectal ulcers, and neoplastic lesions [12,14,17]. PAI^-1^ is involved in tissue preservation and wound repair [34,35]. The overexpression of PAI^-1^ in response to ABS observed in the present study is an initial clue to set further experiments to search the importance of fibrinolysis regulators in the biological effects of ABS. PAI^-1^ is also involved in tumor responses [39,40,41]. In a case series topical ABS inhibited tumor angiogenesis [20], prothrombotic affect of PAI^-1^ in this respect shall also be further searched based on the observation in this study. 

In conclusion, ABS exhibited dual de novo affects on EPCR and PAI^-1^ in HUVECs. HUVECs exposed to LPS challenge caused ABS-induced up-regulation of the expres-sion of EPCR and PAI^-1^, indicating that ABS might act as a topical biological response modifier. As ABS is currently being developed in basic and clinical grounds, these novel observations indicate that additional research on the pleiotropic effects of this unique hemostatic agent is warranted. 

**Acknowledgement**

None of the authors have any conflicts of interest related to the materials used or data presented herein. ABS was obtained from Ankaferd Drug Inc., Istanbul, Turkey.
